# Ca^2+^-Induced PRE-NMR Changes in the Troponin Complex Reveal the Possessive Nature of the Cardiac Isoform for Its Regulatory Switch

**DOI:** 10.1371/journal.pone.0112976

**Published:** 2014-11-13

**Authors:** Nicole M. Cordina, Chu K. Liew, Phani R. Potluri, Paul M. Curmi, Piotr G. Fajer, Timothy M. Logan, Joel P. Mackay, Louise J. Brown

**Affiliations:** 1 Department of Chemistry and Biomolecular Sciences, Macquarie University, Sydney, New South Wales, Australia; 2 Department of Molecular Cardiology and Biophysics, The Victor Chang Cardiac Research Institute, Darlinghurst, New South Wales, Australia; 3 School of Physics, The University of New South Wales, Sydney, New South Wales, Australia; 4 Institute of Molecular Biophysics, Florida State University, Tallahassee, Florida, United States of America; 5 School of Molecular and Microbial Biosciences, University of Sydney, Sydney, New South Wales, Australia; MRC National Institute for Medical Research, United Kingdom

## Abstract

The interaction between myosin and actin in cardiac muscle, modulated by the calcium (Ca^2+^) sensor Troponin complex (Tn), is a complex process which is yet to be fully resolved at the molecular level. Our understanding of how the binding of Ca^2+^ triggers conformational changes within Tn that are subsequently propagated through the contractile apparatus to initiate muscle activation is hampered by a lack of an atomic structure for the Ca^2+^-free state of the cardiac isoform. We have used paramagnetic relaxation enhancement (PRE)-NMR to obtain a description of the Ca^2+^-free state of cardiac Tn by describing the movement of key regions of the troponin I (cTnI) subunit upon the release of Ca^2+^ from Troponin C (cTnC). Site-directed spin-labeling was used to position paramagnetic spin labels in cTnI and the changes in the interaction between cTnI and cTnC subunits were then mapped by PRE-NMR. The functionally important regions of cTnI targeted in this study included the cTnC-binding N-region (cTnI57), the inhibitory region (cTnI143), and two sites on the regulatory switch region (cTnI151 and cTnI159). Comparison of ^1^H-^15^N-TROSY spectra of Ca^2+^-bound and free states for the spin labeled cTnC-cTnI binary constructs demonstrated the release and modest movement of the cTnI switch region (∼10 Å) away from the hydrophobic N-lobe of troponin C (cTnC) upon the removal of Ca^2+^. Our data supports a model where the non-bound regulatory switch region of cTnI is highly flexible in the absence of Ca^2+^ but remains in close vicinity to cTnC. We speculate that the close proximity of TnI to TnC in the cardiac complex is favourable for increasing the frequency of collisions between the N-lobe of cTnC and the regulatory switch region, counterbalancing the reduction in collision probability that results from the incomplete opening of the N-lobe of TnC that is unique to the cardiac isoform.

## Introduction

Troponin (Tn) is a large (∼77 kDa), dynamic protein complex that is located on the thin filament of striated muscle and is responsible for controlling the interaction of myosin with actin. Contraction in striated muscle (cardiac and skeletal) is initiated by the simple binding of calcium (Ca^2+^) to Tn which then initiates a series of conformational changes throughout the protein complex that successively alter protein-protein interactions between actin and tropomyosin in the thin filament, leading to muscle contraction [1], [2]. Despite a wealth of structural data on Tn and its subunits from both x-ray crystallography and nuclear magnetic resonance (NMR), definition of the molecular details of the conformational changes triggered by Ca^2+^ binding within the intact Tn complex is still lacking and remains a challenge experimentally. This is particularly true for the cardiac Tn isoform (cTn) for which we only have a single incomplete crystal structure of the Ca^2+^ saturated core complex [3].

**Figure 1:**
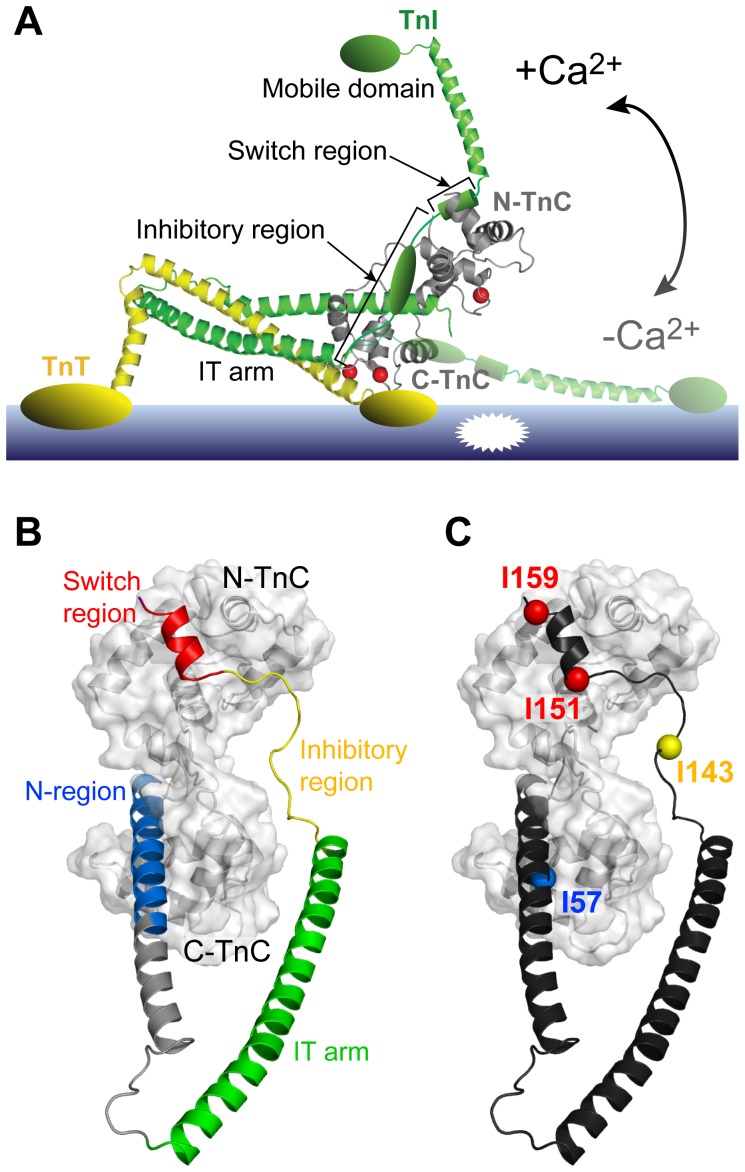
Spin-labeled constructs designed to examine Ca^2+^ induced structural changes in troponin. (A) Structural model of the cTn switch mechanism for thin filament Ca^2+^-mediated activation based on the crystal structure of the core cardiac Tn complex (PDB 1J1D [3]). Ca^2+^ binding to the regulatory domain of the cTnC subunit (grey) triggers a large conformational change, moving the cTnI inhibitory region away from the thin filament and relieving the acto-myosin inhibition (+Ca^2+^ state). (B) Functional regions of cTnI (N-region: blue, IT-arm: green, inhibitory region: yellow, and switch region: red) with cTnC (colored grey, surface representation) in the binary cTnC-cTnI complex (PDB 1J1D [3]). The N-terminal region (blue), inhibitory region (yellow) and the switch region (red) are labeled. Note that the inhibitory region (residues 137–148 of cTnI) is not present in the crystal structures of cardiac Tn [3]; a possible conformation of the missing region is shown here for clarity. (C) Location of residues mutated to cysteine for attachment of spin labels on cardiac TnI: I57 (N-region), I143 (inhibitory region), I151 or I159 (switch region).

The troponin complex (Figure 1A) is composed of three different subunits; the 18 kDa Ca^2+^ binding subunit (TnC), the 24 kDa thin filament binding inhibitory subunit (TnI), and the 35 kDa tropomyosin anchoring subunit (TnT). TnC consists of two globular metal binding domains, the C-lobe and N-lobe, which have structural and regulatory roles, respectively. It is the binding of Ca^2+^ to the regulatory N-lobe that is responsible for initiating contraction. Following Ca^2+^ binding, the signal is propagated from the N-lobe to the TnI subunit and subsequently to the other members of the thin filament (tropomyosin and actin). This cascade of conformational events modifies the interaction between the actin thin filament and the myosin thick filament, leading to muscle contraction.

The TnI subunit alone is capable of inhibiting acto-myosin ATPase activity [4], [5], [6]. However, this action of the inhibitory TnI subunit is regulated by Ca^2+^ in the presence of the other Tn subunits of the complex [7], [8]. Under conditions of low Ca^2+^, two regions of TnI (Figure 1A) are reported to interact with the thin filament (reviewed by Li et al 2004 [9]). These are the ‘inhibitory region’ (cardiac residues 137–148) or primary actin binding region of TnI; and the ‘mobile domain’ (cardiac residues 170–210) or secondary actin binding region; although a recent molecular dynamics (MD) study suggests that these roles might be reversed for the cardiac isoform [10]. Separating these two regions of TnI is an amphipathic helix termed the ‘switch region’ (cardiac residues 150–159). The current model for muscle regulation posits that Ca^2+^ binding promotes the opening of the regulatory N-lobe of TnC to reveal a hydrophobic pocket which allows for binding of the TnI switch region [11], [12]. It is this interaction of the switch region with TnC that is also required to stabilise the ‘open’ conformation of the N-lobe of the cardiac isoform of TnC [13]. Binding of the switch region to the N-lobe of TnC then leads to the disruption of the thin filament interactions, allowing for the acto-myosin interaction (Figure 1A).

A crystal structure of a 52 kDa cardiac Tn core complex (cTn), comprising cTnC (residues 1–161), cTnI (residues 31–210), and cTnT (182–288), was solved in the presence of Ca^2+^ in 2003 [3] (Figure 1B). Two years later, the crystal structure of the core complex for the skeletal isoform (skTn) in the high Ca^2+^ state, as well as a low resolution (7 Å) partial structure in the Ca^2+^-free state, were also determined [14]. The crystal structures of the Ca^2+^-bound Tn core complex for both isoforms showed Tn to have two distinct sub-domains. The first, called the structural ‘IT-arm’ domain (Figure 1A), contains a long coiled-coil region from the C-terminal TnT fragment intertwined with the N-terminal regions of TnI and the structural C-lobe of TnC. The second ‘regulatory domain’ consists of the TnC N-lobe regulatory domain and the C-terminal regions of TnI. However, notably absent in the structure of the crystallized cardiac isoform were a number of functionally important regions involved in the Ca^2+^-switch mechanism. Firstly, both the cardiac specific cTnI N-extension region (residues 1–30), which can be phosphorylated *in vivo* [13], [15], and the N-terminal domain of cTnT (1–181), which tethers the complex to the thin filament, were removed to aid crystallisation. Secondly, insufficient electron density prevented determination of the structure of most of the cTnI inhibitory region (137–146) and a large portion of the cTnI C-terminus mobile region (residues 192–210, Figure 1A). Lastly, the central linker of cTnC connecting the two metal binding lobes was similarly disordered and thus poorly defined in the cardiac crystal structure [3].

Nevertheless, the Ca^2+^-bound cTn and skTn core crystal structures did provide confirmation of binding of the TnI switch region to the hydrophobic pocket of the N-lobe of TnC, which was also observed by fluorescence resonance energy transfer (FRET) experiments [16], [17] and NMR studies of skTnI peptides complexed with truncated skTnC constructs [18], [19]. Additionally, the crystal structures of the skeletal isoform demonstrated the complete release of the switch region from the N-lobe of skTnC upon the removal of Ca^2+^ [14]. However, the position of the switch region upon release still remains poorly defined, as only the first two amino acids of the skeletal switch region are visualised in the −Ca^2+^ crystal structure, suggesting a highly dynamic region. Despite a decade now passing since these crystal structures were reported, the structure of the cardiac isoform in the absence of Ca^2+^ has remained elusive. Without the ability to compare structural models of Ca^2+^-bound and free states of cardiac Tn, questions have continued to exist over whether the movements suggested by the crystal structures even occur in muscle cells. Indeed, there is still no direct evidence collected in solution using full-length Tn subunits that support the large scale movement of TnI away from TnC for the cardiac isoform. Therefore, a more detailed structural understanding of the Ca^2+^ regulation mechanism for the release of the switch region from cTnC is needed.

Here we have used paramagnetic relaxation enhancement (PRE) NMR spectroscopy to further understand the Ca^2+^-controlled conformational interplay between the key functional regions of TnI with TnC in the cardiac isoform. PRE-NMR extends the capabilities of traditional NMR approaches to probe protein structure and dynamics by providing long-range distance information up to 30 Å for a proton experiencing relaxation enhancement from a nearby paramagnetic moiety, such as a nitroxide spin label [20]. These long-range constraints can then be used to spatially map intermolecular binding surfaces and position elements within macromolecular complexes [21], [22], [23]. To map the interactions between cTnI-cTnC, nitroxide spin labels were strategically positioned on the intact troponin binary complex (cTnC-cTnI) with the goal of tracking the Ca^2+^ sensitive movement of several regions of cTnI (Figure 1C). The regions of cTnI targeted for spin label incorporation included the functionally important switch and inhibitory regions, as well as the N-region located in the IT-arm (Figure 1B). Four spin-labeled variants of full-length cTnI were generated and reconstituted with full length cTnC. PRE NMR experiments were then performed on each construct in the absence and presence of Ca^2+^ in order to define the position of these key regions of cTnI with respect to full-length cTnC. Our results support a model of the cardiac isoform where the cTnI switch peptide is clearly released from the N-lobe of cTnC in the low Ca^2+^ state; however, upon release, it remains in close vicinity to cTnC. We speculate that the close proximity of cTnI to cTnC is favourable for increasing the frequency of collisions between cTnC and the switch region, counteracting the reduced probability of collisions due to the incomplete opening of the N-lobe of cTnC in the cardiac isoform [11].

## Materials and Methods

### Cardiac Tn constructs

Cysteine-less constructs of rat cardiac TnC (cTnC) and rat cardiac TnI (cTnI) were expressed in the pET-3d expression vector (Novagen) as previously described [24]. Four mono-cysteine cTnI constructs were generated from the cysteine-less cTnI construct using the QuikChange II site-directed mutagenesis kit (Stratagene), with the introduced cysteine residues located at selected positions along the cTnI sequence. These residues were cTnI58 (N-region), cTnI143 (inhibitory region); and cTnI151 and cTnI159 (switch region). All constructs were verified by DNA sequencing and transformed into *Escherichia coli* BL21 (DE3) cells for expression.

### Expression and purification of cTnI

Monocysteine constructs of cTnI were expressed in Terrific Broth medium. Cell lysis was achieved with 2–3 passes through a chilled French Press at 10,000 psi. The insoluble cTnI inclusion bodies were separated from soluble proteins in the cell lysate by centrifugation (30,966× g, 20 mins, 4°C). The inclusion bodies were solubilised via dialysis into a buffer containing 6 M urea, 1 mM DTT, 1 mM EDTA and 20 mM Tris, pH 8.0, and Complete EDTA-free Protease Inhibitors (Roche). After overnight dialysis, the sample was clarified by centrifugation (30,966× g, 20 mins, 4°C). The supernatant was loaded onto a 1.5 cm×15 cm carboxymethyl (CM) Sepharose column (GE Healthcare). cTnI was eluted with a linear NaCl gradient from 0 M NaCl to 0.3 M NaCl, as previously described [24]. Fractions containing pure cTnI were identified with SDS-PAGE.

### [^15^N] labeling of cTnC

Cysteine-less cTnC was expressed in M9 minimal media as described by Sambrook *et al* (1989) [25], containing 2.5 g/L glucose and 1 g/L ^15^NH_4_Cl, using the IPTG induction protocol of Studier *et al* (1990) [26]. The ^15^N-cTnC was then purified by hydrophobic interaction chromatography using a 1.5 cm×15 cm Phenyl Sepharose 6 Fast Flow column (GE Healthcare), as previously described [27]. cTnC fractions identified by SDS-PAGE were pooled before further purification on a 2.5 cm×7.5 cm DEAE-Sephadex A25 column (Sigma-Aldrich). Pure cTnC fractions eluted at ∼0.5 M KCl and were concentrated using an Amicon Ultra-15 centrifugal filtration device.

### Paramagnetic labeling of cTnI with MTSL

The paramagnetic nitroxide moiety was introduced onto the sulfhydryl group of the cysteine side-chain of each mono-cysteine cTnI construct by the covalent attachment of the spin label 1-oxyl-2,2,5,5-tetra-methylpyrroline-3-methyl-16 methanethiosulfonate (MTSL) (Toronto Research Chemicals). Immediately prior to labeling, the purified cTnI constructs (∼7 mg/mL) were incubated with 20 mM DTT (dithiothreitol) for 2 h at 4°C to ensure complete reduction of the single cysteine sulfhydryl group. DTT was then removed from the sample using a 5 mL HiTrap desalting column (GE Healthcare) and the reduced cTnI was eluted in labeling buffer (6 M urea, 100 mM KCl, 1 mM EDTA, 50 mM MOPS pH 7.9). A 7.5 fold molar excess of MTSL was added immediately to the reduced cTnI. After 2 h incubation at 4°C, an additional 2.5 fold excess of MTSL was added to the sample before overnight incubation at 4°C with gentle stirring. Excess MTSL was then removed by exhaustive dialysis against 2 M urea, 1 mM EDTA, 100 mM KCl, 50 mM MOPS pH 7, before dialyzing into reconstitution buffer (6 M urea, 1 mM EDTA, 0.5 M KCl, 2 mM CaCl_2_, 3 mM MgCl_2_, 50 mM MOPS pH 7.2).

EPR spectroscopy was used to determine the spin-labeling efficiency of each MTSL-labeled cTnI sample. All EPR experiments were performed on a Bruker EMX X-Band (9.5 GHz) spectrometer using a standard rectangular TE cavity at room temperature. First derivative EPR absorption spectra were collected with a microwave power of 5.0 mW, modulation amplitude of 1.0 G and sweep width of 140 G. EPR spectra were analyzed with a LabView package (National Instruments) of EPR spectral analysis programs. Spin labeling yields for each sample were determined from the integrated area under the EPR absorbance spectrum by interpolation from a calibration curve constructed from MTSL standards. Complete modification with MTSL (>95%) was achieved for all cTnI mutant constructs.

### Reconstitution of binary complex samples for PRE-NMR (^15^N-cTnC/MTSL-cTnI)

The reconstituted binary ^15^N-cTnC/MTSL-cTnI constructs are referred to according to the position of the spin-labeled cTnI residue (I57, I143, I151 or I159; Figure 1C). Binary complex formation was achieved by incubating ^15^N-cTnC with each of our four MTSL-labeled ^14^N-MTSL-cTnI constructs in a 4∶5 molar ratio in reconstitution buffer containing 6 M urea and 0.5 M KCl. Urea was first removed by dialysis before the concentration of KCl was lowered to 0.1 M over a 12 hour period using a linear KCl gradient to precipitate excess cTnI. After the removal of precipitated protein by centrifugation (12,000× g for 20 minutes), complex formation was verified using SDS-PAGE and size exclusion chromatography (SEC) on a Superdex 75 10/300 GL column (GE Healthcare).

NMR binary samples in the +Ca^2+^ state were prepared by exhaustive dialysis into NMR buffer (200 mM KCl, 20 mM imidazole pH 6.9) containing 3 mM CaCl_2_. To prepare calcium free samples (−Ca^2+^), Ca^2+^ was removed by dialysis against 0.1 M EGTA for 2 h at 4°C followed by exhaustive dialysis into decalcified NMR buffer (treated with Chelex-100 resin, Bio-Rad) with 5 mM MgCl_2_, as previously described [11].

### PRE-NMR spectroscopy of binary complex (^15^N-cTnC/MTSL-cTnI)

NMR data were collected on a Bruker Avance 600 MHz spectrometer equipped with a cryoprobe. All experiments were performed at 303 K and spectra were processed using Topspin 1.3 (Bruker, Inc). All NMR samples were 550 µL, and concentrations of the binary complex (cTnC-cTnI) ranged from 150 µM to 250 µM. These low concentrations were required to prevent intermolecular PRE effects [28]. For paramagnetic spin-labeled samples, ^1^H-^15^N-TROSY spectra were collected for each of the four (I57, I143, I151, I159) spin labeled binary ^15^N-cTnC/MTSL-cTnI samples. After acquiring each paramagnetic spectrum, the nitroxyl radical of the MTSL label was reduced to its hydroxylamine equivalent by the addition of ∼1/250 volume of a 5-fold molar excess of ascorbic acid (from a 0.5 M stock) directly to the NMR sample. One hour at room temperature was sufficient time to allow for the reduction reaction to go to completion as confirmed by EPR. ^1^H-^15^N-TROSY spectra were then collected for each reduced diamagnetic sample. The small volume of ascorbic acid did not significantly dilute the sample, thus enabling the direct comparison of peak intensities between paramagnetic and diamagnetic spectra for calculation of the relaxation enhancement. Spectral assignment and analysis was performed with the program Sparky [29]. Peak assignments in the diamagnetic and paramagnetic spectra of each cTnC construct were made using the assignments reported for the cardiac binary complex (cTnC-cTnI) [30], and using the chemical shifts of isolated cTnC in the Ca^2+^-free state [11].

### PRE data analysis

The PRE caused by the MTSL spin label (*Γ_2_*) was calculated from the ratio of peak intensities measured in the paramagnetic state (*I_para_*) and the diamagnetic state (*I_dia_*) for each amide peak assigned in the ^1^H-^15^N-TROSY spectra of the binary constructs (Eqn. 1). Peak heights were used as measures of peak intensities, and the intrinsic transverse relaxation rate (*R_2_*) was estimated from the peak width at half-height (*R_2_ = πΔv_1/2_*) [31], [32]. All spectra were recorded with the single evolution time point (*t*) of 10 ms.
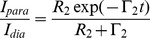
(1)


The calculated PRE rates (*Γ_2_*) were converted to distances (*r*) using a modified form of the Solomon-Bloembergen equation [31], [32], [33]:
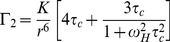
(2)where *ω_H_* is the Larmor frequency of the proton; *K* is a constant (1.23×10^−32^ cm^6^ s^−2^) which describes the spin properties of the nitroxide spin label [32]; and *τ_c_* is the correlation time of the electron-proton interaction [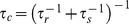]. Due to the long electronic relaxation time of the nitroxide spin label (*τ_s_*) compared to the protein rotational correlation time (*τ_r_*), the value of *τ_c_* is effectively equal to the overall correlation time of the protein [34] and was taken as 16 ns, as estimated from linewidths. Due to the steep distance dependence of the PRE effect, the *τ_c_* value used in Eqn. 2 has a relatively minor impact on distance determinations; with a 2 ns uncertainty in *τ_c_* causing, at most, <6% change in the PRE calculated distance [32]. It should be noted that in order to convert experimental *Γ_2_* values to distances using Eqn. 2 we assume that the observed PRE effects arise from a single conformation.

The distances measured within cTnC-cTnI were compared to the crystal structure of the cardiac troponin core obtained in high Ca^2+^ conditions [3]. Distances from the Cα atom of the spin labeled cTnI residues to the backbone amide groups of cTnC residues were calculated from the coordinates in the PDB 1J1D [3].

## Results

### Preparation of paramagnetic labeled cTn binary samples for PRE-NMR

The goal of this study was to perform PRE measurements in the intact 42.6 kDa cTnC-cTnI binary cardiac complex in solution, in order to track the movement of key regions of cTnI with respect to cTnC in response to Ca^2+^ binding. Four sites on cTnI were selected for spin labeling and the paramagnetic label attached via introduced cysteine residues (Figure 1C). These sites included two positions on the regulatory switch region (I151 and I159), one in the inhibitory region (I143), and one in the cTnI N-region (I57). Of these four label sites, only residue I143 is unresolved in the crystal structure of the +Ca^2+^ cardiac Tn core complex. Each cTnI construct was labeled with the paramagnetic spin label (MTSL) with >95% efficiency, as judged by double integration of the EPR spectrum. To obtain the intersubunit PRE-NMR constraints, cysteine-less cTnC was isotopically labeled with ^15^N and the four binary cTnC-cTnI complexes were prepared by individually reconstituting each spin-labeled cTnI construct with the ^15^N-cTnC protein. Binary complex formation was confirmed by SDS-PAGE and analytical size exclusion chromatography (Figure S1).

### PRE effects in +Ca^2+^ and −Ca^2+^ ^15^N-TROSY spectra of the cTnC-cTnI binary complex

**Figure 2:**
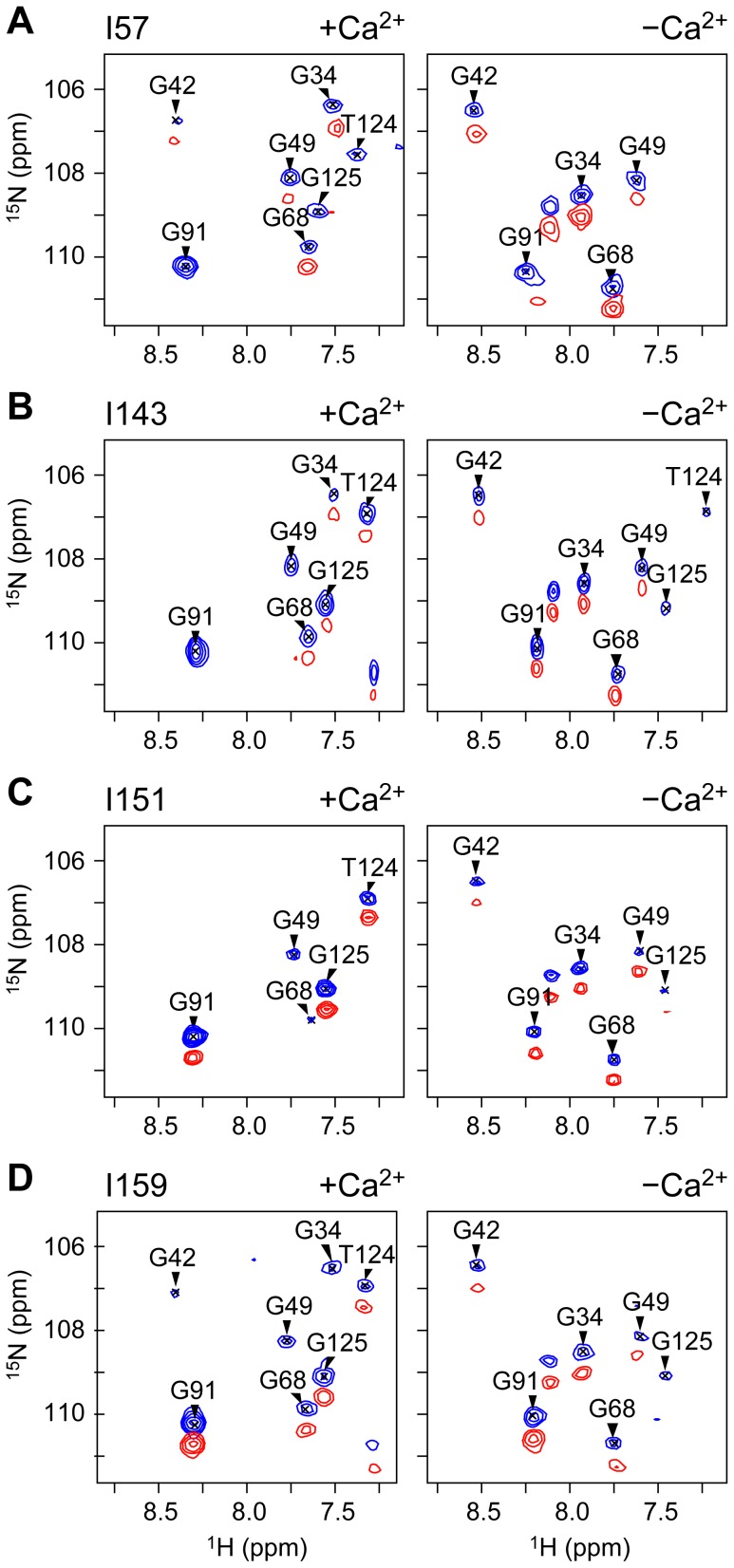
Strong peak broadening due to the PRE effect was observed in cTnC residues in the vicinity of each cTnI spin label. The glycine-containing region of the paramagnetic (red) and diamagnetic (blue) ^1^H-^15^N-TROSY spectrum of each spin-labeled binary cTnC-cTnI construct. The MTSL spin label is attached to cTnI residues (A) I57, (B) I143, (C) I151 and, (D) I159. Spectra are shown for +Ca^2+^ (left panels) and −Ca^2+^ conditions (right panels). The superimposed paramagnetic spectrum is offset by 0.5 ppm in the ^15^N dimension for clarity.

NMR samples of each spin-labeled binary cTnC-cTnI construct were prepared in both the absence of calcium (−Ca^2+^), and presence of Ca^2+^ (+Ca^2+^). A ^1^H-^15^N-TROSY spectrum was first acquired for each sample in the presence of the paramagnetic spin label (Figure S2, Figure 2). A second spectrum was then acquired following the reduction of the nitroxide group of the spin label to its diamagnetic equivalent. In each case, peak broadening/disappearance was observed in the paramagnetic spectrum and was alleviated in the diamagnetic spectrum.

Assignment of all four +Ca^2+^ ^1^H-^15^N-TROSY spectra (Figure 2) was made by comparison with available chemical shift values of cTnC bound to cTnI(1–211) [30] and cTnI(1–80) [35]. Using this approach, 40–50% of cTnC residues could be assigned with confidence in the +Ca^2+^ spectra of each construct. A comparable number of peak assignments was achieved for the four −Ca^2+^ spectra using the chemical shifts of isolated cTnC N-domain resonances in the Ca^2+^-free state [11]; 32–52% of cTnC resonance peaks in the −Ca^2+^ spectra were assigned with confidence. The assigned peaks for both the +Ca^2+^ and −Ca^2+^ states for all four spin labeled constructs provided good overall coverage of the N- and C-domains of cTnC, as well as the connecting central linker region (cTnC residues 85–93).

PRE effects are observed in each of our binary constructs as peak broadening and reduction in peak intensity in the paramagnetic state (*I_para_*/*I_dia_*<1). The PRE-broadening of the NMR signal is proportional to *1/r^6^* (Equation 2), where *r* is the distance between the proton and the nitroxide group of the spin label. At very short distances, resonance peaks in the paramagnetic ^1^H-^15^N-TROSY become broadened beyond detection. The shortest distance measurable using the PRE method is therefore dependant on the noise level in the spectrum. The upper limit is also dependent on the noise level in the spectrum, since the changes in peak intensity must be detectable above the noise contribution to peak intensity. In ideal cases, very small changes in peak intensity may be detectable for resonances located at distances of up to 30 Å from the spin label (for small proteins, such as the isolated 18 kDa cTnC subunit [11], [27]). Increased spectral noise is an unavoidable complication in the spectra of large proteins; such as the 42.6 kDa cTnC-cTnI binary complex here. Due to the noise level in ^1^H-^15^N-TROSY spectra of this complex, an estimated uncertainty of 5% in our peak intensity ratios limited the range of quantifiable PRE effects to 12–25 Å.

### Changes in cTnC peak intensity in the cTnC-cTnI complex in response to Ca^2+^ binding

**Figure 3:**
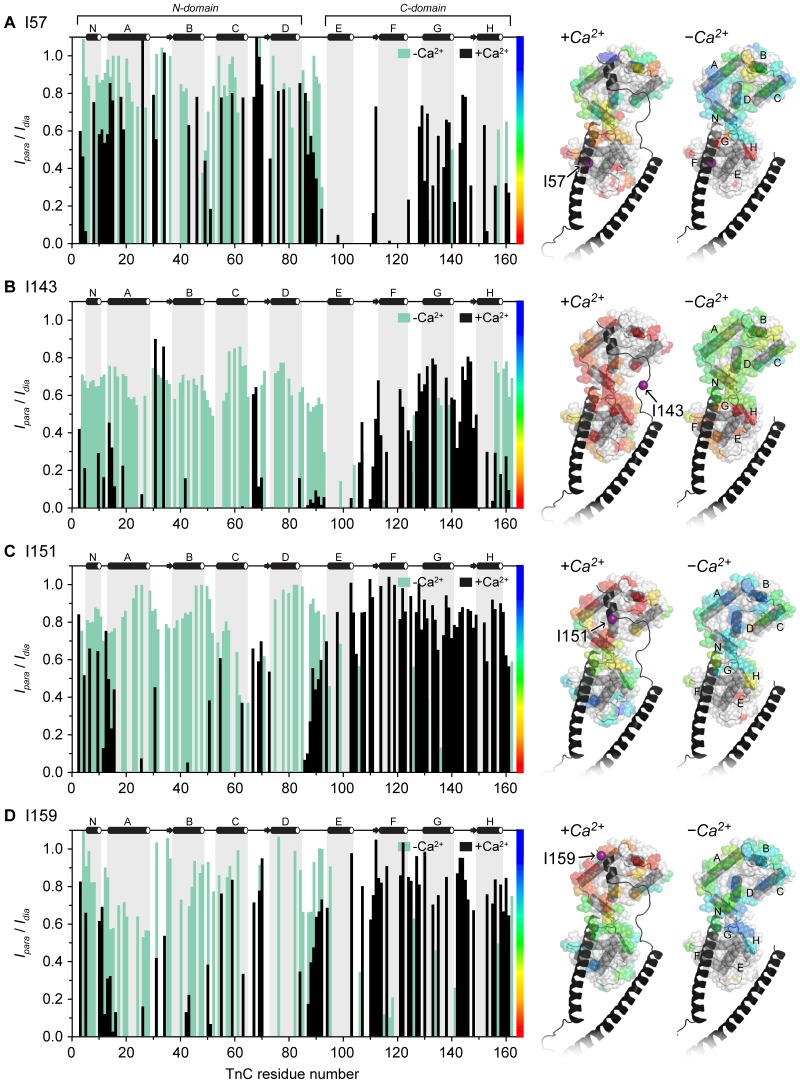
PRE-NMR peak intensity ratios (*I_para_/I_dia_*) mapped onto cardiac Tn in the presence and absence of Ca^2+^. Four monocysteine cTnI constructs were spin-labeled with MTSL at residues (A) I57, (B) I143, (C) I151 and (D) I159. The cTnI spin labels caused distance dependant peak broadening for ^15^N-cTnC residues in ^1^H-^15^N-TROSY spectra of the cTnC-cTnI complexes. The magnitude of the peak broadening is determined by comparing the paramagnetic peak intensity (*I_para_*) to the peak intensity in the diamagnetic state (*I_dia_*) after the reduction of the MTSL spin label. *Left:* Peak intensity ratios (*I_para_/I_dia_*) for each assigned cTnC residue, in the absence of Ca^2+^ (green) and presence of Ca^2+^ (black). Peaks that were broadened beyond detection are assigned a peak intensity ratio of 0.05. *Right:* the peak intensity ratios are mapped onto the crystal structure of cardiac Tn (1J1D [3]) according to the scale bar, where red indicates a large reduction in peak intensity and blue indicates no change. cTnI is colored black, and the location of the spin labeled cTnI residue is indicated with a purple sphere and an arrow. cTnC helices N, A–H are labeled.

For all four binary complex samples, a unique pattern of paramagnetic peak broadening effects arising from the inclusion of the spin label on cTnI were observed (Figure 2). Peak intensity ratios (*I_para_/I_dia_*) calculated for each assigned cTnC resonance in the −Ca^2+^ and +Ca^2+^ are plotted in Figure 3. The peak intensity ratio values are also mapped onto the +Ca^2+^ cardiac Tn crystal structure (1J1D) in a continuous color scale. Strong PRE peak broadening effects (*I_para_/I_dia_*≪1) are shaded red and are indicative of close proximity (<12 Å) of the cTnC residue to the nitroxide spin label on cTnI. cTnC resonances which were unaffected by the spin label (*I_para_/I_dia_*∼1) are shaded in blue and represent residues >25 Å away from the spin label. cTnC residues for which no peak intensity ratio could be reliably assigned, either due to uncertainty in assignment or peak overlap, are shown in white. This comparative mapping of the PRE broadening effects enables the rapid visualisation of the relative positioning of each of the functional cTnI regions under investigation with respect to cTnC. The mapping also reveals the effect of Ca^2+^ binding on the relative positioning of the two subunits.

### The N-region of cTnI remains tightly bound to C-lobe of cTnC in the absence of Ca^2+^

Comparison of the profiles of peak intensity ratios obtained in the absence and presence of Ca^2+^ show that the spin label on I57 (cTnI N-region) had a similar effect on cTnC residues in both Ca^2+^ states (Figure 3A). Strong broadening effects were most notable for cTnC C-lobe residues (*I_para_/I_dia_*<0.4, as mapped in red/yellow). This indicates that the position of the N-region of cTnI (Figure 1B) is in close proximity to the structural C-lobe of cTnC, and that they remain tightly bound to each other in both the absence and presence of Ca^2+^.

Also notable in the I57 data is that many residues in the regulatory N-lobe of cTnC are broadened by the presence of the spin label. This observation is in agreement with the conclusion of our earlier PRE study which showed that the N- and C-lobes form an overall compact conformation in solution [27]. In the binary cTnC-cTnI complex, the effect of the I57 spin label on cTnC N-lobe resonances is slightly weaker upon the removal of Ca^2+^. This suggests that in the Ca^2+^-free state, the N-lobe of cTnC is slightly further from the I57 site (and thus the C-lobe of cTnC, where the N-region of cTnI is tightly bound). This movement of the two lobes of cTnC relative to one another could result from an increase in dynamics of the central linker, a change in the interdomain angle of cTnC towards a more extended conformation, or a combination of both. Ca^2+^ associated changes in intermolecular cTnC PRE effects were also observed for the other three spin labeled sites (Figure 3B–D).

### In the Ca^2+^-free state, the switch region is released from the hydrophobic N-lobe of cTnC

While the local effects of the I57 spin label site are quite similar in the +Ca^2+^ and −Ca^2+^ states, substantially different PRE effects were observed between the +Ca^2+^ and −Ca^2+^ states for the other three spin label positions. In the presence of Ca^2+^, discrete patches of strong PRE effects (*I_para_/I_dia_*<0.4, red/yellow) were observed for cTnC N-lobe residues in the complexes with the spin label in the switch region (I151 and I159) (Figure 3C–D). This well-defined patch of strong PRE effects that map to the N-lobe of cTnC for both the I151 and I159 constructs indicate that the switch region is tightly bound within the hydrophobic pocket of the regulatory N-lobe in the +Ca^2+^ state. Upon the removal of Ca^2+^, a significant reduction in PRE broadening was observed for N-lobe residues (*I_para_/I_dia_*>0.5, green/blue), indicating release of the switch peptide from the N-lobe of cTnC. We did not observe any other localised region of strong PRE broadening upon release of the switch region in the −Ca^2+^ state (Figure 3C–D). It is therefore unlikely that the switch region binds to an alternate site on cTnC in the Ca^2+^-free state. Rather, upon release from the hydrophobic N-lobe, the switch peptide probably undergoes dynamics that places it near the cTnC C-lobe for some of the time, thus accounting for the moderate PRE-broadening effects observed for several residues in the C-domain.

### The inhibitory region is highly dynamic and close to cTnC

The most dispersed and largest Ca^2+^-induced differences in PRE effects on cTnC residues were observed for the spin label attached to I143 on the inhibitory region of cTnI (Figure 3B). The spin label at this site caused strong relaxation enhancements under high Ca^2+^ conditions for a broad but defined spatial region on cTnC, centred on the linker region between the N-domain and C-domain (Figure 3B). This suggests that, unlike the switch region, the inhibitory region is not tightly bound to cTnC at a well-defined location in the presence of Ca^2+^. Rather, the position of this region is highly variable. These PRE effects are consistent with a model where the inhibitory region is a mobile element that is tethered upstream (through the interaction of the switch region with the N-lobe of cTnC), and downstream (by the N-region of cTnI through its interaction with C-lobe of cTnC). In the absence of Ca^2+^, the effect of the I143 spin label was considerably weaker for a large number of residues throughout the N-lobe and central linker of cTnC. In the C-lobe, there was little difference in the PRE effects observed for most residues in the +Ca^2+^ and −Ca^2+^ states, except for helix H, for which considerably stronger PRE effects were observed in the +Ca^2+^ state. Together, these observations suggest that upon removal of Ca^2+^, the inhibitory region still remains in close proximity to cTnC through its downstream interaction with the C-lobe of cTnC (shown by label at I57), but due to the release of the switch region from the cTnC N-lobe (as shown by labels at I151 and I159), the inhibitory region samples a much larger range of conformations in the Ca^2+^-free state.

### Ca^2+^ regulated movement of the cardiac switch region

**Figure 4:**
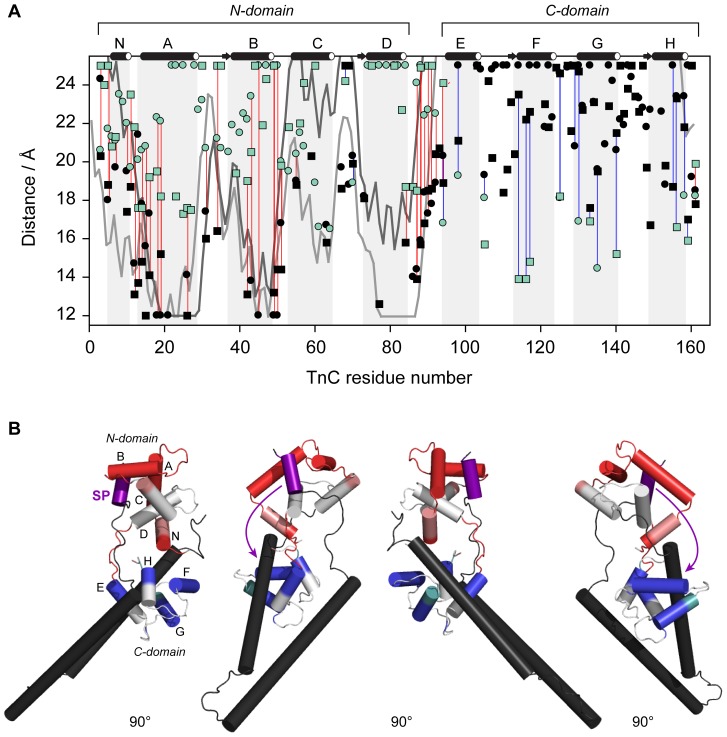
Distances between cTnC residues and the switch region probe sites cTnI151 (circles) and cTnII159 (squares). Distances measured in the absence of Ca^2+^ (green) are compared to those measured in the presence of Ca^2+^ (black). For reference, the distances calculated from the co-ordinates of the cardiac Tn crystal structure 1J1D are also shown (I151: light grey line, I159: dark grey line line). Paired measurements (listed in Table S1 and Table S2) are highlighted with a blue line in cases where *r*[+Ca^2+^]>*r*[−Ca^2+^] or a red line where *r*[+Ca^2+^]<*r*[−Ca^2+^]. The helices of cTnC are indicated with light grey shading (N, A–D in the N-domain, and E–H in the C-domain). The distances changes are mapped onto the cardiac Tn crystal structure (PDB 1J1D); red coloring where *r*[+Ca^2+^]<*r*[−Ca^2+^], and blue when *r*[+Ca^2+^]>*r*[−Ca^2+^]. Direction of movement of the switch region (purple) upon release of Ca^2+^, suggested by the changes in distances, is indicated with arrows in (B). The distance changes are consistent with the movement of the switch region toward the C-domain.

The mapping of the PRE broadening patterns for label sites I151 and I159 suggested the tight binding of the switch region to the N-lobe under high Ca^2+^ conditions and its release upon removal of Ca^2+^. Peak intensity ratios were then used to calculate the PRE rates (*Γ*_2_) (Eqn. 1) for the switch region constructs I51 and I159 (Figure S3). Distances can then be derived from PRE rates by application of the Solomon-Bloembergen equation (Eqn. 2). Comparison of distances measured from the switch region sites of cTnI to cTnC in the crystal structure of the cardiac Tn core in the presence of Ca^2+^ (1J1D) were in very good agreement (Figure 4), confirming that in solution, the switch region is indeed locked into the hydrophobic pocket of the N-lobe under high Ca^2+^ conditions.

Comparison of the distances measured in the +Ca^2+^ and −Ca^2+^ states for I151 and I159 reveal the Ca^2+^-dependent direction of movement of the switch region. These distance changes between the +Ca^2+^ and −Ca^2+^ states are also mapped onto the cardiac Tn core crystal structure (1J1D, [3]) in Figure 4B. Helices of cTnC colored red display a decrease in distance when Ca^2+^ is removed (*r*[+Ca^2+^]>*r*[−Ca^2+^]) indicating an increase in the average distance between the spin label and the cTnC residue. Similarly, blue coloring indicates an increase in distance between the spin label and cTnC when Ca^2+^ is removed.

Compared to the Ca^2+^-bound state, the distances measured in the Ca^2+^-free state are substantially longer for N-lobe helices A, B, and D. Slightly longer distances were also observed for cTnC residues located in the defunct Ca^2+^ site I between helices A and B (residues 28–40), and the central linker region (residues 85–93) of cTnC, indicating that upon release of Ca^2+^, the switch region moves away from these regions of cTnC. Conversely, distances for the C-domain helices F and G (and to a lesser extent, helix H) are longer in the −Ca^2+^ state, indicating that upon release from the N-lobe in the Ca^2+^-free state, the switch region moves towards these helices in the C-lobe of cTnC.

All distances measured from the switch region (I151 and I159) to cTnC residues that were able to be assigned in both the +Ca^2+^ and −Ca^2+^ states are listed in Table S1 and Table S2, respectively. The difference between these distance values reveals the magnitude of movement that the switch undergoes upon release. The average distances measured from the switch region to cTnC residues located in helix B (centre of the N-lobe) and helix F (centre of the C-lobe) in the absence and presence of Ca^2+^ are further given in Table 1. Upon release from the N-domain, the switch region moves ∼10 Å away from the N-domain and ∼10 Å closer to the C-domain. It should be noted that the distances obtained from the PRE rates (and summarised in Table 1) assume a single conformational state for the switch region. This is a reasonable assumption in the +Ca^2+^ state since the switch region is tightly bound to the N-domain of cTnC. However in the Ca^2+^-free state, our data suggests that the switch region is not bound to cTnC, thus the interpretation of the distances measured in the −Ca^2+^ state should be treated with caution.

**Table 1:** PRE-NMR distances between cTnI switch region and cTnC domains.

*cTnC region*	1J1D 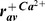	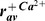	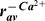	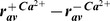 (Å)
N-domain (helix B)	15.3	13.0	22.5	−9.5
C-domain (helix F)	43.5	23.3	13.7	9.7

The average distances (Å) measured from the cTnI switch region (I151 and I159) to cTnC residues located in helix B (centre of the N-lobe) and helix F (centre of the C-lobe), in the absence and presence of Ca^2+^. The average distance calculated from the coordinates of the cardiac Tn crystal structure in the +Ca^2+^ state (1J1D [3]) are also included in the table.

## Discussion

We have used PRE to follow the conformational changes associated with binding of Ca^2+^ to troponin within the binary cTnC-cTnI complex. Using the site-specific placement of nitroxide spin labels in cTn, we have mapped the quaternary interactions and described the change in average distance between key regions of the cTnI inhibitory subunit and the cTnC Ca^2+^ binding subunit. Our PRE-NMR studies clearly show that the switch peptide is tightly bound within the N-lobe of cTnC in the presence of Ca^2+^, whereas the inhibitory region exhibits conformational freedom, but remains in the vicinity of the central linker region of cTnC. In the absence of Ca^2+^, the switch peptide is completely released from the N-lobe of cTnC. Upon release, it moves ∼10 Å towards the C-lobe of cTnC. However, we see no evidence of an alternative binding site on cTnC for the switch peptide in the Ca^2+^-free state. Our PRE measurements also show that the interaction of the N-region of cTnI with the structural C-lobe of cTnC is, unsurprisingly, Ca^2+^-independent.

The geometrical positioning of key functional regions of the TnI subunit with respect to TnC and the subsequent movement of TnI accompanying activation, for both skeletal and cardiac isoforms, has been the subject of many biochemical studies over the past two decades [19], [36], [37], [38]. From these studies, structural models describing the cascade of conformational changes arising from binding of Ca^2+^ to the N-lobe of cTnC have been proposed with the molecular details for the positioning of most functional regions of cTnI reinforced by the crystal structure of the cardiac Tn core structure made available in 2003 [3]. However, definitive supportive data, required to provide a more complete detailed description of these structural models and associated Ca^2+^-induced changes, is lacking, since there is no crystal structure of the cardiac complex in the low Ca^2+^ state. Determination of such a structure is likely to remain a significant challenge due to its dynamic nature.

### Obtaining a high-resolution structure of the Ca^2+^-free cTn complex

While other NMR studies have also been performed on the complete binary cTnC-cTnI complex in the +Ca^2+^ state [30], [35], [39], here we report for the first time that NMR-derived distances can be measured between subunits of the complete binary complex in both the Ca^2+^-bound and Ca^2+^-free states. To perform PRE-NMR in the cTnC-cTnI binary complex required the spin label to be placed on ^14^N-cTnI and reconstituted with ^15^N-cTnC. This was necessary in order to simplify our ^1^H-^15^N-TROSY spectra, since it has previously been demonstrated that skTnI resonance peaks are poorly dispersed, as is typical for partially disordered proteins [40]. While cTnI was not directly observed in our ^1^H-^15^N-TROSY spectra, the PRE broadening effect caused by the cTnI spin labels enabled us to indirectly monitor the functionally important regions of cTnI in the binary complex using full-length cTnC and full-length cTnI. Using this approach in the 42 kDa binary complex, we have identified the regions of cTnI that are most sensitive to Ca^2+^ binding. Sufficient inter-subunit measurements were obtained to describe the magnitude of movement, or lack of movement, for each of the cTnI regions upon removal of Ca^2+^.

Overall, our PRE experiments showed no large scale structural rearrangement of the two cTn subunits with respect to each other; only the selective and modest movement of the switch and the inhibitory region were observed. The PRE broadening patterns for the two labels on the switch region (Figure 3C–D) showed that in the presence of Ca^2+^, the cTnI switch region interacts with a small, well-defined small region of cTnC, consistent with tight binding of this region to the N-lobe of cTnC. This was in agreement with the binding mode of the switch region in the crystal structure of cardiac Tn [3] (Figure S3) and the solution structure of the N-lobe of cTnC in complex with the cTnI fragment of the switch region reported by Li et al [41].

### The inhibitory region does not interact tightly with cTnC

The PRE broadening effects for the spin label on the inhibitory region were observed to map to a large, but well-defined area of cTnC centred on the central linker. The inhibitory region is likely held in close proximity to the central linker of cTnC as a result of being tethered at either end by the strong interactions between the N-region of cTnI with the C-lobe of cTnC, and the switch region with N-lobe of cTnC. The broad region over which the strong PREs were observed also suggests that this region is highly mobile in solution. Lindhout et al [42] identified electrostatic interactions between the basic residues within the inhibitory region of a cardiac TnI fragment (residues cTnI R141, R145, R146 and R148) and acidic patches centred on the C-lobe of cTnC (E-helix) and the central linker. While our results indicate that the inhibitory region is not tightly bound to cTnC, due to the close proximity of the basic inhibitory region and the acidic cTnC residues, such transient electrostatic interactions between these regions are possible.

### In the cardiac isoform, the switch region of cTnI remains in close vicinity to cTnC

A key goal of our study was to map changes in the interaction between the cTnI and cTnC subunits upon the removal of Ca^2+^, in particular the positioning of the cTnI switch region upon release from cTnC. The magnitude of movement we observed for the switch region upon release from the N-domain in the Ca^2+^-free state for both label sites was only modest. The average distance change measured between the switch peptide and cTnC was only up to ∼10 Å (Table 1). A similar magnitude of movement of the switch region is also reported to occur in reconstituted thin filaments for the cardiac isoform [43], [44]. In this FRET study, the authors reported the switch region (labeled at cTnI150) moves only ∼6.5 Å further away from the central helix of cTnC (labeled at 89) in the low Ca^2+^ state compared to the high Ca^2+^ state. From our cTnI151 label, we observed a similar distance change of 4 Å. Further support for the lack of any large movement of the switch region was recently demonstrated in a MD simulation study of the cardiac Tn isoform using 45 FRET distance constraints [10]. A network of hydrogen bonds was observed between the switch region and helices B and C of cTnC, consistent with features of our PRE mapping profile. Furthermore, the authors reported in their MD study that the switch region in the Ca^2+^-free state ‘was still held’ and ‘did not drop off’ the cTnC N-domain hydrophobic pocket. They proposed that upon release of the switch region from the N-domain of cTnC, the modest movement could arise due to the folding and collapse of the D/E helices upon release of Ca^2+^ and the switch region from the N-domain in the Ca^2+^-free state. Jayasundar *et al* report a distance change of 5.5 Å between cTnI151 and cTnC, similar to our measured distance change [10]. If the small shift in the position of the switch region, as observed in our study and the FRET study, is indeed responsible for transmitting the initial Ca^2+^ binding signal from the N-lobe of cTnC to the other components of the thin filament, then the switch region is likely to already be in close proximity to the thin filament in the activated state. Positioning of these key domains of cTnI close to the actin filament can only occur if the central linker of cTnC is flexible such that the N-lobe remains in proximity to the thin filament at all times, in contrast to current Ca^2+^ regulation models (Figure 1A). We and others have shown that although cardiac TnC has considerable interdomain flexibility, it still preferentially adopts a compact conformation in solution under saturating Ca^2+^ conditions, with a defined range of relative domain orientations [27].

The PRE data in our previous study also suggested the presence of significant interdomain contacts between the two lobes of cTnC, supporting a collapsed conformation for the cardiac Tn complex. Others have also proposed that the central linker adopts an even more flexible conformation once in the Ca^2+^-free state, which would bring the switch region even closer in proximity to the actin filament [10]. However we could not confirm this with our data. Regardless, the likely bent conformation of cTnC results in the N-lobe being in close proximity to the thin filament so that upon the release of the switch region from the N-lobe of cTnC, the switch region needs only move a short distance towards the thin filament to allow for the interaction of the primary and secondary actin binding regions of cTnI to inhibit the acto-myosin interaction.

Of interest is a recent EPR study reporting inter-spin distance measurements to monitor the release of the switch region in reconstituted thin filaments in the skeletal Tn isoform [45]. In contrast to our findings, the authors reported an average increase of >40 Å in the distance between the switch region and N-lobe of skTnC upon the removal of Ca^2+^. Isoform differences in the nature of the conformation of TnC may provide an explanation for the longer distance measured for the skeletal isoform. Indeed, the authors attributed this large increase in distance between the switch region and the N-lobe of TnC in the skeletal isoform by proposing that the N-lobe of skTnC becomes more mobile in the Ca^2+^-free state due to melting of the central helix from an extended conformation in the +Ca^2+^ state which is presumably further away from the thin filament in the high Ca^2+^ state. Large structural changes in the intact skeletal Tn ternary complex on Ca^2+^ release have also been observed using small-angle neutron and x-ray scattering [46]. This significant difference between the two studies of the Tn isoforms reinforce the importance of continuing to independently decipher the mechanism of regulation of contraction for the cardiac and skeletal isoforms.

The close proximity of the switch region to TnC in the cardiac isoform upon removal of Ca^2+^ may also be explained by the altered Ca^2+^-response of the cardiac TnC isoform. Unlike its skeletal counterpart, Ca^2+^-binding to does not fully open the N-lobe of cTnC, rather, the N-lobe exists in an equilibrium of closed and open states [13], [35], [47]. We have recently shown that Ca^2+^-binding to isolated cTnC results in an increase in the population of the open state to ∼27% [11]. The probability of the cTnI switch peptide colliding with cTnC in a suitable binding mode (‘open’) is therefore considerably less likely for cardiac TnI, compared to skeletal TnI. However, as we have shown here, the switch region of cTnI remains close to the N-lobe of cTnC at all times and therefore collisions should occur at a higher frequency for the cardiac isoform.

The results presented in this work demonstrate that the site-directed spin-labeling PRE NMR approach carries great promise for constructing accurate three dimensional models of whole troponin and providing a complete description of the dynamics of the intact complex. Positioning of spin labels at additional optimal sites, as suggested by this initial mapping of the interactions of cTnC with cTnI, offers a viable strategy to continue to map the transient tertiary and quaternary interactions in the cTn system which are fundamental for understanding the Ca^2+^ mediated regulation of cardiac muscle contraction.

## Supporting Information

Figure S1 **Purification of the binary cTnC-cTnI complex.** (A) SDS-PAGE (12.5% acrylamide) was used to assess the purity of the preparation of binary samples for NMR. Samples of purified cTnI (Lane 1: *I*) and cTnC (Lane 2: *C*) were reconstituted as a binary cTnC-cTnI complex (Lane 3: *I+C*). (B) NMR samples of binary cTnC-cTnI were monomeric and homogenous, as judged by analytical size exclusion chromatography (SEC) on a Superdex 75 10/300 GL (GE Healthcare) column. Samples of the binary cTnC-cTnI complex (black line) were found to consist of a single species of MW of ∼42 kDa which was significantly greater than that of isolated cTnC of ∼18 kDa (grey). NMR buffer (3 mM CaCl_2_, 200 mM KCl, 20 mM imidazole pH 6.9) was used for all analytical SEC runs.
(DOCX)

Figure S2 **Two dimensional ^1^H-^15^N-TROSY spectra of binary troponin complexes.** MTSL spin label attached to cTnI residues: I57-MTSL in the (A) +Ca^2+^ and (B) −Ca^2+^conditions; I143 in the (C) +Ca^2+^ and (D) −Ca^2+^conditions; I151-MTSL in the (E) +Ca^2+^ and (F) −Ca^2+^conditions; and, I159 in the (G) +Ca^2+^ and (H) −Ca^2+^conditions. The paramagnetic spectrum (*red*) is superimposed on diamagnetic (*blue*) ^1^H-^15^N-TROSY spectrum after reduction of the spin labels for each sample.
(DOCX)

Figure S3 **PRE rates (Γ2) in cTnC residues caused by the switch region probe site cTnI151 (circles) and cTnII159 (squares).** PRE rates measured in the absence of Ca^2+^ (green) are compared to those measured in the presence of Ca^2+^ (black). The helices of cTnC are indicated with light grey shading (N, A–D in the N-domain, and E–H in the C-domain).
(DOCX)

Table S1 **PRE-NMR distances between cTnI switch (cTnI151) and cTnC.** Distances measured from the cTnI151 spin label to cTnC residues, for residues which were measurable in both the +Ca^2+^ and −Ca^2+^ states. The highlighted distances were used to calculate the averages values presented in Table 1.
(DOCX)

Table S2 **PRE-NMR distances between cTnI switch (cTnI159) and cTnC.** Distances measured from the cTnI159 spin label to cTnC residues, for residues which were measurable in both the +Ca^2+^ and −Ca^2+^ states. The highlighted distances were used to calculate the averages values presented in Table 1.
(DOCX)
