# Supplementary material for: Ca2+-Induced PRE-NMR Changes in the Troponin Complex Reveal the Possessive Nature of the Cardiac Isoform for Its Regulatory Switch
Source: PLoS One. 2014 Nov 13;9(11):e112976. doi: 10.1371/journal.pone.0112976 (PMC4231091; doi:10.1371/journal.pone.0112976)
Supplement: Figure S1 — Purification of the binary cTnC-cTnI complex. (A) SDS-PAGE (12.5% acrylamide) was used to assess the purity of the preparation of binary samples for NMR. Samples of purified cTnI (Lane 1: I) and cTnC (Lane 2: C) were reconstituted as a binary cTnC-cTnI complex (Lane 3: I+C). (B) NMR samples of binary cTnC-cTnI were monomeric and homogenous, as judged by analytical size exclusion chromatography (SEC) on a Superdex 75 10/300 GL (GE Healthcare) column. Samples of the binary cTnC-cTnI complex (black line) were found to consist of a single species of MW of ∼42 kDa which was significantly greater than that of isolated cTnC of ∼18 kDa (grey). NMR buffer (3 mM CaCl2, 200 mM KCl, 20 mM imidazole pH 6.9) was used for all analytical SEC runs. (DOCX) [file pone.0112976.s001.docx]

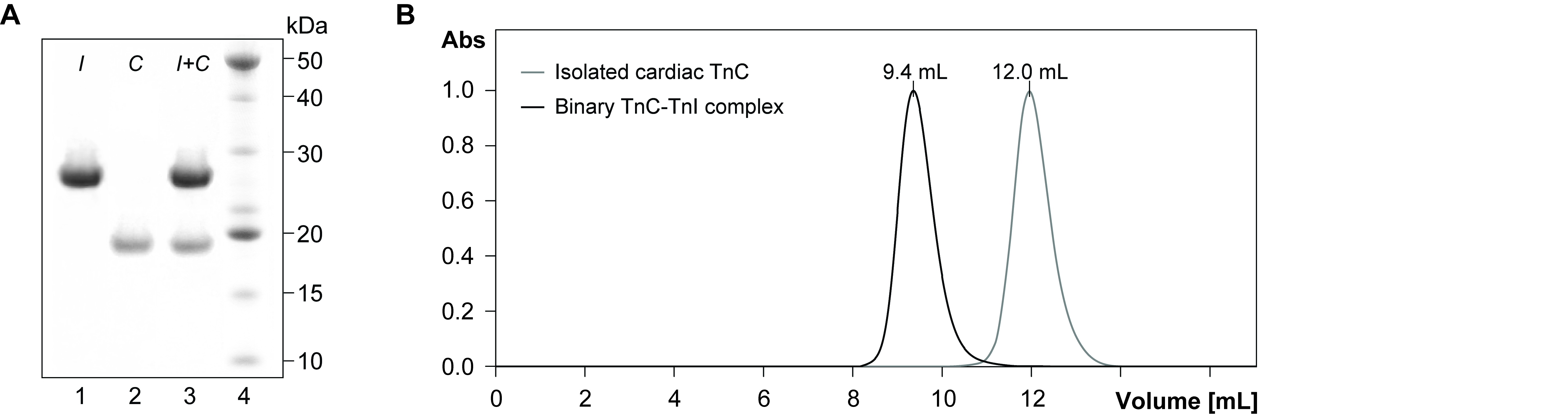


**Figure S1. Purification of the binary cTnC-cTnI complex.** (A) SDS-PAGE (12.5% acrylamide) was used to assess the purity of the preparation of binary samples for NMR. Samples of purified cTnI (Lane 1: *I*) and cTnC (Lane 2: *C*) were reconstituted as a binary cTnC-cTnI complex (Lane 3: *I + C*). (B) NMR samples of binary cTnC-cTnI were monomeric and homogenous, as judged by analytical size exclusion chromatography (SEC) on a Superdex 75 10/300 GL (GE Healthcare) column. Samples of the binary cTnC-cTnI complex (black line) were found to consist of a single species of MW of ~42 kDa which was significantly greater than that of isolated cTnC of ~18 kDa (grey). NMR buffer (3 mM CaCl_2_, 200 mM KCl, 20 mM imidazole pH 6.9) was used for all analytical SEC runs.
