# Supplementary material for: Ca2+-Induced PRE-NMR Changes in the Troponin Complex Reveal the Possessive Nature of the Cardiac Isoform for Its Regulatory Switch
Source: PLoS One. 2014 Nov 13;9(11):e112976. doi: 10.1371/journal.pone.0112976 (PMC4231091; doi:10.1371/journal.pone.0112976)
Supplement: Figure S2 — Two dimensional 1H-15N-TROSY spectra of binary troponin complexes. MTSL spin label attached to cTnI residues: I57-MTSL in the (A) +Ca2+ and (B) −Ca2+conditions; I143 in the (C) +Ca2+ and (D) −Ca2+conditions; I151-MTSL in the (E) +Ca2+ and (F) −Ca2+conditions; and, I159 in the (G) +Ca2+ and (H) −Ca2+conditions. The paramagnetic spectrum (red) is superimposed on diamagnetic (blue) 1H-15N-TROSY spectrum after reduction of the spin labels for each sample. (DOCX) [file pone.0112976.s002.docx]

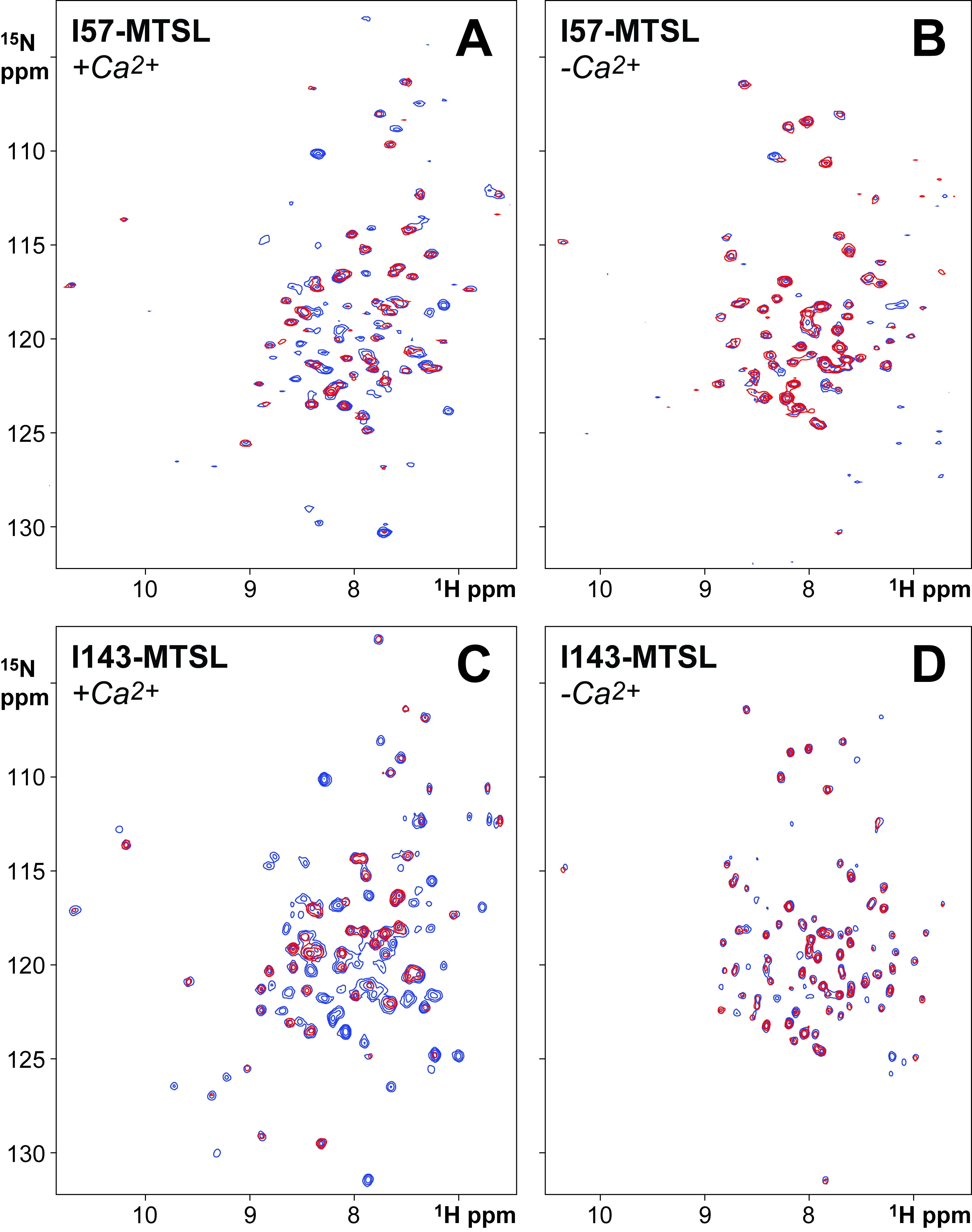


**Figure S2. Two dimensional ^1^H^15^N-TROSY spectra of binary troponin complexes.** MTSL spin label attached to cTnI residues: I57-MTSL in the (A) +Ca^2+^ and (B) -Ca^2+^conditions; I143 in the (C) +Ca^2+^ and (D) –Ca^2+^conditions; I151-MTSL in the (E) +Ca^2+^ and (F) -Ca^2+^conditions; and, I159 in the (G) +Ca^2+^ and (H) –Ca^2+^conditions. The paramagnetic spectrum (*red*) is superimposed on diamagnetic (*blue*) TROSY spectrum after reduction of the spin labels for each sample (*continued over page*).


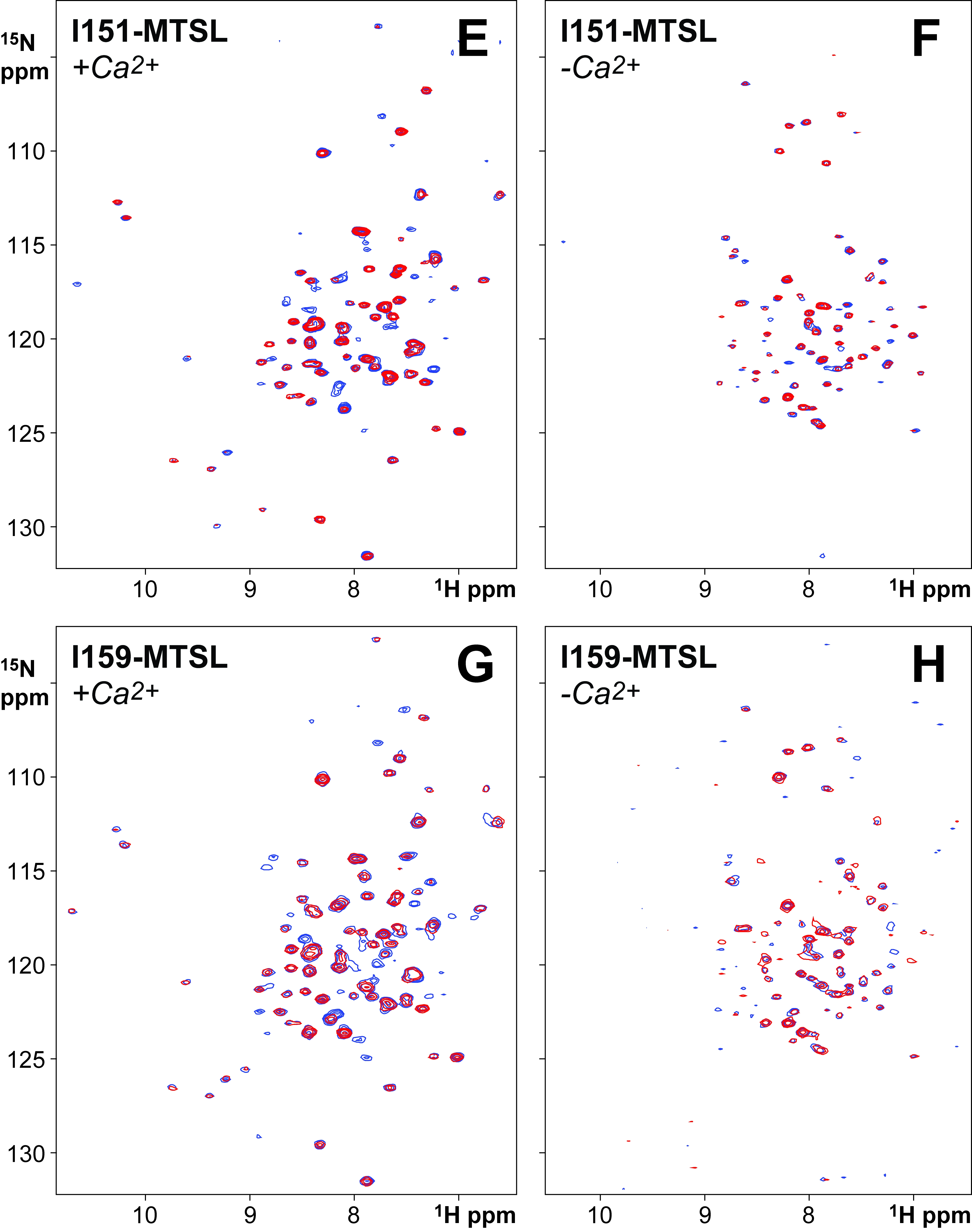


**Figure S2 (*continued*)**
