# Supplementary material for: Ca2+-Induced PRE-NMR Changes in the Troponin Complex Reveal the Possessive Nature of the Cardiac Isoform for Its Regulatory Switch
Source: PLoS One. 2014 Nov 13;9(11):e112976. doi: 10.1371/journal.pone.0112976 (PMC4231091; doi:10.1371/journal.pone.0112976)
Supplement: Figure S3 — PRE rates (Γ2) in cTnC residues caused by the switch region probe site cTnI151 (circles) and cTnII159 (squares). PRE rates measured in the absence of Ca2+ (green) are compared to those measured in the presence of Ca2+ (black). The helices of cTnC are indicated with light grey shading (N, A–D in the N-domain, and E–H in the C-domain). (DOCX) [file pone.0112976.s003.docx]

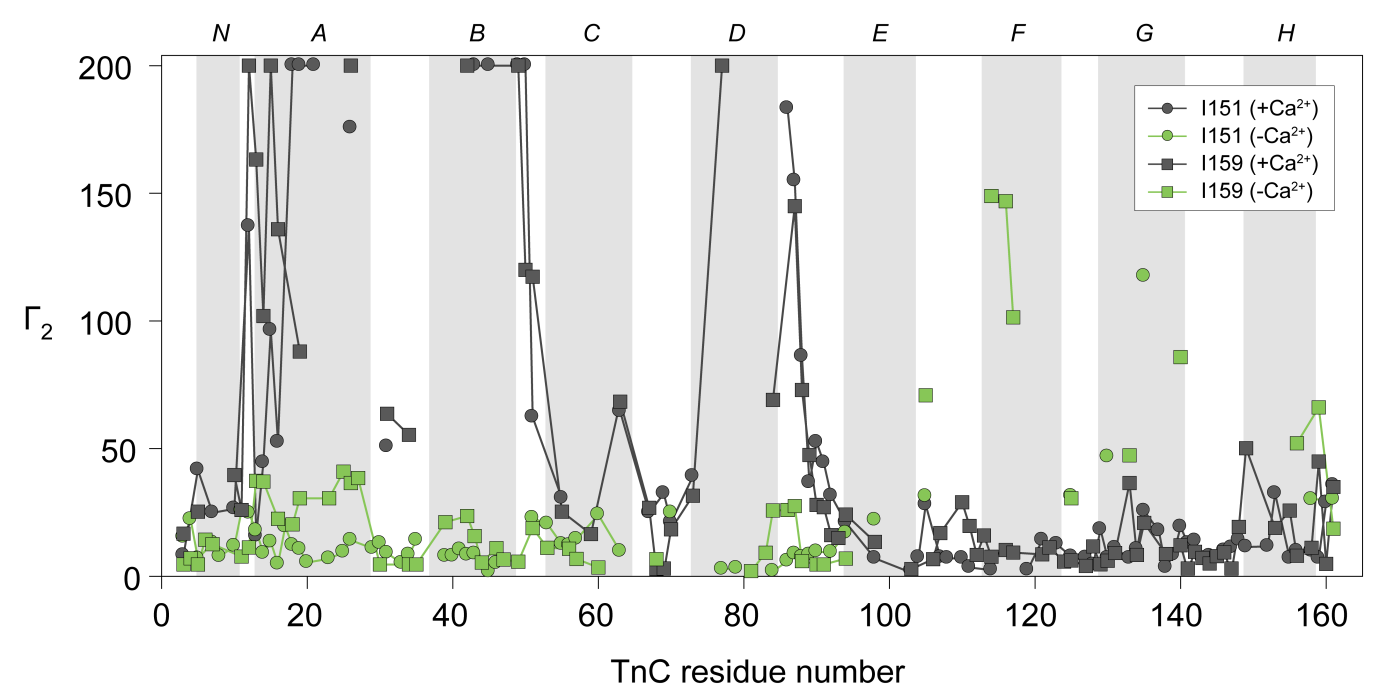


**Figure S3. PRE rates (Γ2) in cTnC residues caused by the switch region probe site cTnI151 (circles) and cTnII159 (squares).** PRE rates measured in the absence of Ca^2+^ (green) are compared to those measured in the presence of Ca^2+^ (black). The helices of cTnC are indicated with light grey shading (N, A-D in the N-domain, and E-H in the C-domain).
