# Supplementary material for: Ca2+-Induced PRE-NMR Changes in the Troponin Complex Reveal the Possessive Nature of the Cardiac Isoform for Its Regulatory Switch
Source: PLoS One. 2014 Nov 13;9(11):e112976. doi: 10.1371/journal.pone.0112976 (PMC4231091; doi:10.1371/journal.pone.0112976)
Supplement: Table S1 — PRE-NMR distances between cTnI switch (cTnI151) and cTnC. Distances measured from the cTnI151 spin label to cTnC residues, for residues which were measurable in both the +Ca2+ and −Ca2+ states. The highlighted distances were used to calculate the averages values presented in Table 1. (DOCX) [file pone.0112976.s004.docx]

| *Residue* | *1J1D* | *I151 (+Ca^2+^)* | *I151 (-Ca^2+^)* | *I151 [+Ca^2+^]- [Ca^2+^]* | *Location* |
| --- | --- | --- | --- | --- | --- |
| 3 | 19.6 | 24.3 | 20.6 | 3.7 |  |
| 5 | 15.7 | 18.0 | 21.7 | -3.7 | N-helix |
| 7 | 17.3 | 19.7 | 21.1 | -1.4 | N-helix |
| 10 | 16.0 | 19.5 | 22.0 | -2.5 | N-helix |
| 14 | 16.8 | 17.8 | 20.6 | -2.8 | A-helix |
| 15 | 15.9 | 15.6 | 20.8 | -5.2 | A-helix |
| 18 | 13.8 | <12 | 22.3 | -10.3 | A-helix |
| 19 | 12.4 | <12 | 22.1 | -10.1 | A-helix |
| 31 | 20.9 | 17.4 | 20.7 | -3.3 | Site I |
| 43 | 15.6 | 13.8 | 23.4 | -9.6 | B-helix |
| 45 | <12 | <12 | >25 | -13 | B-helix |
| 49 |  | <12 | >25 | -13 |  |
| 50 |  | <12 | >25 | -13 |  |
| 51 | 16.2 | 16.8 | 20.0 | -3.2 |  |
| 55 | 21.3 | 19.0 | 20.4 | -1.4 | C helix |
| 70 | 21.6 | 20.3 | 18.9 | 1.4 | Site II |
| 87 | 12.0 | 14.4 | 24.1 | -9.7 |  |
| 88 | 13.0 | 15.9 | >25 | -9.1 |  |
| 89 | 15.3 | 18.4 | 22.4 | -4 |  |
| 90 | 18.0 | 17.3 | 22.7 | -5.4 |  |
| 92 | 23.8 | 18.9 | 22.5 | -3.6 |  |
| 94 | 28.8 | 20.3 | 16.8 | 3.5 | E-helix |
| 98 | 32.8 | >25 | 14.0 | 11 | E-helix |
| 114 | 40.3 | >25 | <12 | 13 | F-helix |
| 125 | 34.8 | 24.6 | 18.1 | 6.5 |  |
| 127 | 34.3 | 24.9 | 18.4 | 6.5 |  |
| 128 | 35.8 | >25 | 18.4 | 6.6 |  |
| 130 | 37.5 | >25 | 17.9 | 7.1 | G-helix |
| 155 | 28.6 | >25 | 19.0 | 6 | H-helix |
| 158 | 24.3 | 23.4 | 18.2 | 5.2 | H-helix |
| 161 | 22.0 | 18.5 | 17.9 | 0.6 |  |

**Table S1. PRE-NMR distances between cTnI switch (cTnI151) and cTnC.** Distances measured from the cTnI151 spin label to cTnC residues, for residues which were measurable in both the +Ca^2+^ and -Ca^2+^ states. The highlighted distances were used to calculate the averages values presented in Table I.
