# Supplementary material for: Ca2+-Induced PRE-NMR Changes in the Troponin Complex Reveal the Possessive Nature of the Cardiac Isoform for Its Regulatory Switch
Source: PLoS One. 2014 Nov 13;9(11):e112976. doi: 10.1371/journal.pone.0112976 (PMC4231091; doi:10.1371/journal.pone.0112976)
Supplement: Table S2 — PRE-NMR distances between cTnI switch (cTnI159) and cTnC. Distances measured from the cTnI159 spin label to cTnC residues, for residues which were measurable in both the +Ca2+ and −Ca2+ states. The highlighted distances were used to calculate the averages values presented in Table 1. (DOCX) [file pone.0112976.s005.docx]

| *Residue* | *1J1D* | *I159 (+Ca^2+^)* | *I159 (-Ca^2+^)* | *I159 [+Ca^2+^]- [Ca^2+^]* | *Location* |
| --- | --- | --- | --- | --- | --- |
| 3 | 28.9 | 20.3 | >25 | -4.7 |  |
| 5 | 25.1 | 18.8 | >25 | -6.2 | N-helix |
| 11 | 22.1 | 18.7 | 23.5 | -4.8 | N-helix |
| 12 | 19.5 | 13.1 | 21.8 | -8.7 |  |
| 13 | 18.0 | 13.7 | 17.6 | -3.9 |  |
| 14 | 18.1 | 14.8 | 17.6 | -2.8 | A-helix |
| 16 | 14.2 | 14.1 | 19.2 | -5.1 | A-helix |
| 19 | 12.0 | 15.2 | 18.2 | -3 | A-helix |
| 26 | 12.0 | <12 | 17.6 | -5.6 | A-helix |
| 34 | 19.1 | 16.4 | >25 | -8.6 | Site I |
| 42 | 16.5 | 13.1 | 19 | -5.9 | B-helix |
| 49 | 20.4 | 13.2 | >25 | -11.8 |  |
| 51 |  | 14.4 | 19.8 | -5.4 |  |
| 68 | 27.0 | >25 | 24.2 | 0.8 |  |
| 84 | 17.3 | 15.8 | 18.7 | -2.9 | D-helix |
| 87 | 21.8 | 13.9 | 18.5 | -4.6 |  |
| 88 | 24.3 | 15.7 | 24.9 | -9.2 |  |
| 90 | 29.1 | 18.5 | >25 | -6.5 |  |
| 91 | 31.9 | 18.6 | >25 | -6.4 |  |
| 94 | 40.2 | 18.9 | 24.1 | -5.2 | E-helix |
| 114 | 49.6 | 23.5 | 13.9 | 9.6 | F-helix |
| 116 | 50.8 | 22.2 | 13.9 | 8.3 | F-helix |
| 117 | 48.2 | 22.6 | 14.8 | 7.8 | F-helix |
| 125 | 42.7 | 24.6 | 18.2 | 6.4 |  |
| 140 | 40.1 | 21.5 | 15.2 | 6.3 | G-helix |
| 156 | 38.3 | 23.3 | 16.6 | 6.7 | H-helix |
| 159 | 32.2 | 17.0 | 15.9 | 1.1 |  |
| 161 | 32.3 | 17.8 | 19.9 | -2.1 |  |

**Table S2. PRE-NMR distances between cTnI switch (cTnI159) and cTnC.** Distances measured from the cTnI159 spin label to cTnC residues, for residues which were measurable in both the +Ca^2+^ and -Ca^2+^ states. The highlighted distances were used to calculate the averages values presented in Table I.
